# Long-Term Anatomic and Functional Outcomes after Macular Hole Surgery

**DOI:** 10.1155/2018/3082194

**Published:** 2018-11-26

**Authors:** Karolina Kaźmierczak, Joanna Stafiej, Joanna Stachura, Paweł Żuchowski, Grażyna Malukiewicz

**Affiliations:** ^1^Department of Ophthalmology, Faculty of Medicine, Nicolaus Copernicus University in Toruń, Collegium Medicum, Bydgoszcz, Sklodowskiej-Curie 9, 85-094 Bydgoszcz, Poland; ^2^Clinic of Rheumatology and Connective Tissue Diseases, J. Biziel University Hospital No. 2, Bydgoszcz, Poland

## Abstract

**Aim:**

To evaluate the structural and functional outcomes in patients who underwent macular hole (MH) surgery in the long-term follow-up.

**Materials and Methods:**

Forty-four eyes of 40 patients (28 females and 12 males) were examined. The examination included visual acuity, optical coherence tomography, and colour vision testing. The same evaluation was performed in 30 fellow eyes.

**Results:**

MH closure was obtained in 42 eyes (95.45%). There was no reopening of the initially closed MHs. In long-term postoperative examination, we observed IS/OS junction defects in 28 (63.6%) eyes and ELM defects in 19 (43.2%) eyes. We found that the IS/OS junction defects correlated with the diameter of the MH (*p*=0.016), whereas ELM defects correlated with both the diameter of the MH (*p*=0.001) and duration time of the MH (*p*=0.008). The presence of ELM defects in OCT was the cause of inferior BCVA in long-term observation time (*p*=0.004). The mean BCVA before the MH surgery was 0.15. It improved significantly both in early (*p* < 0.001) and long-term postoperative observation (*p* < 0.005). Generally, the functional outcomes were better in eyes with short-time duration of the MH, when a smaller diameter (<400 *μ*m) of the hole was measured and a V-shaped closure of the MH and the restoration of the ELM line on OCT were present. Pseudoprotanomaly was noted in 13 (35.1%) eyes. In the fellow eye group, mean BCVA was 0.95 (range, 0.6–1.0). In 3 eyes, we detected vitreomacular traction, and in 4 eyes, initial cataract. These conditions, as well as probably early stage of diabetes mellitus, influenced functional outcomes of studied eyes.

**Conclusions:**

The anatomic and functional outcomes after macular surgery are satisfactory and improve with time. After a successful closing of the MH, the restoration of the retina progresses at a slower pace than improvement in visual acuity.

## 1. Introduction

Thanks to the discovery of pathogenesis of the idiopathic full-thickness macular hole by Gass in 1988 [1] and the subsequent development of macular hole surgery, initially introduced by Kelly and Wendel in 1991 [2], adequate treatment of this disease was finally possible. Over the years, a number of modifications in surgical technique were suggested. Peeling the internal limiting membrane (ILM) of the retina was doubtless the most important alteration, which enabled higher anatomic closure and lower reoperation rates in macular holes [3, 4]. Later on, the usage of dyes which improved the visibility of internal limiting membrane (ILM) and development of microsurgery had vastly shortened operation time. However, there are still cases of macular hole where the outcome of surgical repair remains unsatisfactory. One of the main factors influencing postoperative anatomic results was the diameter of the macular hole. Large macular holes were at risk of unclosure. The introduction of the inverted internal limiting membrane flap technique for eyes with large macular holes was an attempt to improve the anatomic success in this group of patients [5]. Although postoperative anatomic success after this technique of surgery was introduced was achieved in most cases, functional improvement compared to conventional peeling was questionable [6]. Recent studies raised the issue of mechanical and subclinical traumatic changes to the retinal nerve fiber layer (RNFL) during macular hole surgery [7, 8]. Furthermore, while the toxicity of indocyanine green (ICG) is well known, the toxicity of other widely used dyes requires further research. These factors may influence the functional outcomes after macular hole surgery.

It is estimated that the risk of developing a macular hole in the fellow eye is less than 2% if posterior vitreous detachment is present, approximately 15% if the posterior vitreous is attached, and as high as 50% should an impending hole be found in the fellow eye [9]. Apart from vitreous attachment condition, little is known of other factors affecting the probability of macular hole formation in the other eye.

The purpose of this study was to evaluate the long-term anatomical and functional outcomes after successful macular hole surgery and to assess the probability of the MH formation in the fellow eye.

## 2. Materials and Methods

Consecutive series of patients undergoing macular hole surgery at the University Hospital in Bydgoszcz, Poland, during 2015 and 2016 were studied prospectively. Only patients with idiopathic full-thickness macular holes (FTMH) in stage 2, 3, or 4 according to Gass were included. Patients with coexisting ocular or systemic diseases, such as diabetic retinopathy, ocular trauma, high myopia, and retinal detachment, were excluded. The cataract phacoemulsification or clear lens phacoaspiration was carried out in all phakic eyes 3 to 5 weeks prior to macular hole surgery to avoid postoperative cataract and postoperative cataract surgery on a vitrectomized eye. One day prior to the pars plana vitrectomy, each patient underwent standard ophthalmologic evaluation, including best-corrected visual acuity (BCVA), using Snellen charts (decimal fraction), slit-lamp biomicroscopy, indirect ophthalmoscopy, fundus photography, and optical coherence tomography (OCT, Nidek RS-3000), as well as colour vision evaluation. The colour vision disturbances were assessed with computerized anomaloscopic methods (Rayleigh and Moreland Colour Matching Tests). To assess diminished red sensitivity, mean values and standard deviations (SD) midmatching points (MMP) were analysed in this study. Early (up to 6 months) and long-term (9–31 months, mean 18 months) postoperative clinical evaluation (using the same examinations as preoperatively) was performed for every patient. Ultimately, 40 patients (44 eyes) were enrolled in the study. Simultaneously, during last follow-up, we measured the advanced glycation end products (AGE) in the skin autofluorescence (SAF), using AGE Reader by Diagnoptics in order to assess the risk of diabetes mellitus development as a possible cause of the dyschromatopsia in fellow eyes without fundoscopic changes and with good visual acuity. Institutional review board approval was obtained.

### 2.1. SAF Measurement Technique

For calculating the risk of cardiovascular diseases (CVD) and diabetes, SAF examination can be conducted [10, 11]. For this purpose, the AGE Reader diagnostic device, manufactured by Diagnoptics, was used.

The patient places his or her forearm onto the device. AGE molecules are then excited through ultraviolet light type B (UVB). The photons initially absorbed by the AGEs are emitted in a process called autofluorescence and are counted by the built-in photomultiplier. The higher the SAF signal, the higher the risk of developing diabetes and CVD by the subject [10–13].

### 2.2. Surgery Technique

Standard 25G pars plana vitrectomy with ILM peeling and C3F8 gas tamponade was performed. During the operation, after core vitrectomy, Brilliant Blue dye was applied on the posterior pole for 30 seconds, ILM peeling was carried out with complete fluid-air exchange and 15% C3F8 endotamponade. In 3 cases of very large macular holes (>700 *μ*m), inverted internal limiting membrane flap was performed. All the operations were performed by the same experienced surgeons. Face-down posturing was recommended for 5 days after operation.

### 2.3. Statistical Analysis

The data obtained are presented as a mean ± standard deviation (SD). For normally and equally distributed data, the groups were compared using a *t*-test. For examination of parameter distribution in the study group, a chi-squared test was used. *p* < 0.05 was considered a statistically significant difference. Statistical analysis was performed using MS Excel 2010.

## 3. Results

Forty-four eyes of 40 patients (28 females and 12 males) who underwent macular hole surgery were enrolled in the study. The mean age at the time of operation was 68.75 ± 6.05 years (range, 58 to 81 years). The mean duration of the macular hole, estimated from visual symptoms reported by the patients, was 8.02 months (range, 2 to 24 months). The visual outcomes in the study group were analysed in terms of short-time (≤6 months) and long-time (>6 months) duration of the macular hole. Regarding duration time, there were 27 (61.4%) eyes and 17 (38.6%) eyes, respectively. Four patients (10%) had a bilateral MH, while 36 (90%) had the unilateral one. The mean diameter of the macular hole was 468.75 *μ*m (range, 140 to 790 *μ*m). Five (11.36%) eyes had a stage-2 macular hole, 17 (38.64%) eyes had a stage 3, and 22 (50%) eyes had a stage 4. Only in eyes with the duration time of symptoms smaller than 6 months, the diameter of the MH was small (≤250 *μ*m). Such a small MH was observed in 5 (11.36%) cases. The mean diameter of the MH in eyes with short-time duration was 397.15 (±154.52) *μ*m, whereas with long-term duration it was 582.47 (±131.5) *μ*m. It was statistically significant that the longer the duration time, the larger the MH (*p*=0.0002). The epiretinal membrane (ERM) was detected in 18 (40.9%) eyes. From the studied group of 44 eyes, the macular hole closure was obtained in 42 eyes (95.45%). There was no reopening of the initially closed macular holes. Apart from one patient, who developed nonarteritic anterior ischaemic optic neuropathy (NAION) in both eyes (one with a macular hole but without a macular hole in the fellow eye) a few months after the operation, we observed no ocular complications in vitrectomized eyes. In 2 eyes, which did not achieve complete closure of the MH, the duration time was 24 months, and the minimum diameter of the MH was more than 600 *μ*m. The baseline characteristics of these patients are presented in Table 1.

The anatomic and functional outcomes after macular hole surgery were evaluated in the study group at 3 time points: at baseline (before surgery), in early postoperative time (≤6 months), and in the long-term follow-up (range 9–31 months, mean 18 months).

### 3.1. Anatomic Outcomes

At baseline, apart from the diameter of the MH, other features in the macular region were clearly present in OCT scans. In 38 (86.3%) eyes, we observed small, yellowish-white deposits on the colour image, also seen on OCT scans as irregularities on the surface of the retinal pigment epithelium (RPE), which correspond to proliferations of the RPE. These findings negatively correlated with preoperative visual acuity (*p*=0.03). Thickened edges of the hole with multiple cystic cavities were observed in 39 (88.6%) eyes and an elevated edge of the hole in 28 (63.6%) eyes.

The postoperative shape of the MH was described as a V-shaped closure, a U-shaped closure, an irregular closure, or a flat/open closure. In terms of the shape of the MH, it was found in 14 (31.8%) eyes, 20 (45.5%) eyes, 4 (9.1%) eyes, and 6 (13.6%) eyes, respectively.

Early after the operation, besides a closed macular hole, some reorganization of the centrofoveal tissue was noted. Residual cystic cavities were seen in 7 (15.9%) eyes. We observed retinal thinning in the foveal region in 11 (25%) eyes. We made every effort to properly evaluate the integrity of hyperreflective lines visible on OCT scans: the photoreceptor inner segment/outer segment (IS/OS) junction and the external limiting membrane (ELM), which proved to be the most important features linked with the improvement of visual acuity. We also attempted to estimate the integrity of the cone outer segment tips (COST) but found it to be most challenging using this type of OCT, related to poorer axial resolution than in SD-OCT. Consequently, we abandoned the attempt to evaluate the COST due to possible adulteration of the outcomes. IS/OS junction defects were found in 35 (79.5%) eyes and ELM defects in 27 (61.4%). Similarly, in long-term postoperative examination, we observed these changes in 28 (63.6%) eyes and 19 (43.2%) eyes, respectively.

We found that the IS/OS junction defects correlated with the diameter of the MH (*p*=0.016), whereas ELM defects correlated with both the diameter of the MH (*p*=0.001) and duration time of the MH (*p*=0.008). The presence of ELM defects in the OCT was the cause of inferior BCVA in long-term observation (*p*=0.004).

We confirmed correlation between IS/OS defects and ELM defects (*p*=0.0001). The IS/OS line had a chance of restoration only if the ELM line was already reconstructed.

Residual cystic cavities were found in 3 (6.8%) eyes and retinal thinning in the foveal region in 14 (31.8%) eyes. The structural outcomes in the study group are presented in Table 2.

### 3.2. Functional Outcomes

#### 3.2.1. Visual Acuity

The mean visual acuity before the MH surgery was 0.15 (median 0.1, SD 0.13, range 0.01–0.5). It has improved significantly both in early (≤6 months) (*p* < 0.001) and long-term (>9 months, range 9–31 months) postoperative observation (*p* < 0.005). In early follow-up, the mean BCVA in eyes with macular closure (42 eyes) was 0.33 (median 0.30, SD 0.24, range 0.01–0.8). In this group, BCVA improved in 32 (76.2%) eyes, was unchanged in 9 (21.4%) eyes, and decreased in 1 (2.4%) eye. Fourteen (33.3%) eyes had a BCVA of 0.5 or more.

In the long-term follow-up, the mean BCVA was 0.50 (median 0.50, SD 0.32, range 0.01–1.0). In this group, BCVA improved in 31 (73.8%) eyes, was unchanged in 6 (14.3%) eyes, and decreased in 5 (11.9%) eyes. In 2 eyes of the same patient, a significant reduction in BCVA occurred a few months after macular hole surgery because of NAION. The improvement in BCVA of 0.5 or more was noted in 27 (64.3%) eyes.

Generally, the functional outcomes were better in eyes with a short-time duration MH, when a smaller diameter (<400 *μ*m) of the hole was measured, when a V-shaped closure of the MH was observed on OCT scans, and when the restoration of the ELM line on the OCT was present. The preoperative and postoperative visual acuity outcomes in eyes with closed macular holes after surgery are summarized in Table 3.

#### 3.2.2. Colour Vision

In this study, we examined colour vision in both axes: red-green and blue-yellow, using computerized anomaloscopic tests (Rayleigh and Moreland Colour Matching Test). Out of all 40 patients, 15 did not attend colour vision testing and 9 had too low BCVA at baseline time point. Therefore, in initial evaluation, we were able to obtain colour vision outcomes in only 19 eyes (16 patients). The mean values and standard deviations (SD) midmatching points (MMP) were analysed. In the Rayleigh equation, the mean MMP was 1.04 (SD 0.11). We observed normal trichromacy (NT) in 14 (73.7%) eyes and pseudoprotanomaly (PPT) in 5 (26.3%) eyes. In the Moreland equation, the mean MMP was 0.94 (SD 0.15). We noted NT in 3 (15.8%) eyes and an achromatic pattern (AchP) in 16 (84.2%) eyes.

At early postoperative time, only 15 patients reported for colour vision testing. In the Rayleigh equation, the mean MMP was 0.98 (SD 0.08). We observed the NT in 11 (73.3%) eyes and PPT in 4 (26.7%) eyes. In the Moreland equation, the mean MMP was 1.0 (SD 0.12). Computer identified the NT in 1 (6.7%) eyes and AchP in 14 (93.3%) eyes.

At the long-term follow-up time point, the colour vision testing was possible in 37 eyes (33 patients). Among other nontested patients, four had not reported for examination and three had insufficient BCVA to undergo the test. In the Rayleigh equation, the mean MMP was 0.97 (SD 0.08). The NT was observed in 24 (64.9%) eyes and PPT in 13 (35.1%) eyes. In the Moreland equation, the mean MMP was 0.99 (SD 0.11). The NT was found in 4 (10.8%) eyes and AchP in 33 (89.2%) eyes. The vision colour outcomes are presented in Table 4.

#### 3.2.3. Fellow Eye

In order to investigate possible changes in the other eye of the patients admitted for macular surgery, we also examined 30 eyes of 30 patients during their last visit. The mean BCVA was 0.95 (range, 0.6–1.0, SD 0.1). In seven eyes, visual acuity was <1.0. In 3 eyes, we detected vitreomacular traction (VMT), and in 4 eyes, initial cataract. These conditions influenced the functional outcomes of studied eyes. Apart from the VMT observed in 3 eyes, we found no other abnormalities in OCT scans. In the Rayleigh equation, the mean MMP was 0.95 (SD 0.08). The NT was observed in 24 (80%) eyes and PPT in 6 (20%) eyes—three of them were diagnosed with VMT and 2 (6.7%) eyes with cataract. In the Moreland equation, the mean MMP was 0.95 (SD 0.12). The NT was found in 17 (56.7%) eyes and AchP in 13 (43.3%) eyes. An achromatic pattern was noted in all 7 eyes, either with VMT or cataract. Six eyes (20%) were completely healthy, with very good visual acuity and without any changes, both in eye fundus examination, as well as OCT examination. We analysed possible causes of the concomitance a good vision and dyschromatopsia. Apart from very rare congenital conditions, which affect selectively S-cones, the most probable reason of such abnormality appears to be a diabetic mellitus (DM) without retinopathy. Among 16 patients with colour disturbances in the fellow eye, the DM was diagnosed in 2 (12.5%). In others, standard laboratory tests such as glucose level were in normal range, but in order to detect an early stage of DM or to assess the risk of DM occurrence, we also performed additional examination of skin autofluorescence (SAF), using AGE Reader by Diagnoptics. Based on the SAF value, there are 4 risk groups: no risk—SAF value was less than or equal to the mean value for the given age range; low risk—SAF value was greater than the mean value for the given age range, but did not exceed 1 standard deviation; increased risk—SAF value exceeded the mean value by more than 1 SD, but it was less than 2.9; and high risk—SAF value was greater than or equal to 2.9. Among 16 examined patients, 9 (56%) had high risk (SAF ≥2.9), 2 (12.5%) increased risk, 3 (18.75%) low risk, and 2 (12.5%) no risk. Apart from 2 diabetic patients, all of the other 6 patients from the study group with dyschromatopsia in fellow healthy eye had high risk of DM development.

## 4. Discussion

An operation on idiopathic macular holes is considered an anatomic success when the MH is closed. The MH closure ratio is high and varies between 85 and 100% [14–16]. This is possible thanks to significant progress in surgical technique, with small-gauge surgery and dye-assisted ILM peeling being critical.

Michalewska et al. introduced the inverted internal limiting membrane flap technique for large macular holes [5]. The authors reported an improvement in both functional and anatomic outcomes of surgery of macular holes with a diameter greater than 400 *μ*m and potential preclusion of postoperative flat-open appearance of a macular hole. Whereas some studies are consistent with these observations [17], others fail to confirm clear superiority of the inverted ILM flap technique in terms of anatomical and functional improvement [6]. In our study, we performed the inverted ILM flap technique in 3 eyes with very large macular holes (>700 *μ*m) and gained MH closure in two of them. On the other hand, we used standard ILM peeling in the other 20 eyes with a large-diameter MH (450–700 *μ*m) and observed complete closure of the MH in all but one eye, which confirms the observation of other authors that the inverted ILM flap technique should be reserved specifically for very large macular holes exceeding 700 *μ*m [18].

In order to predict postoperative functional outcome after macular hole surgery, investigators evaluated such preoperative variables as preoperative visual acuity, duration time of symptoms, and the minimum diameter of the MH measured by OCT or scanning laser ophthalmoscopy [19–21]. Kusuhara et al. suggested the macular hole index (MHI), defined as the ratio of the hole height to the base diameter, which refers to the perpendicular and horizontal dimensions of the hole visualized by cross-sectional OCT images, as a prognostic factor for visual outcome after macular hole surgery [22]. Michalewska et al. presented postoperative outcomes based on the shape of the macular closure (U-shaped closure, V-shaped closure, irregular closure, and flat/open closure) [23]. They suggested that the U-shaped closure, the most common foveal contour, results in the best visual outcomes postoperatively and is present in about 45% of all patients. On the other hand, the V-shaped closure, observed in about 26%, is associated with worse visual outcomes than the U-shaped ones. Irregular closure was reported in about 8.8%, whereas flat/open closure constituted about 19% of cases and was related to poor visual recovery. Our observations are in line with that. In our study, a U-shaped closure was observed in 20 (45.5%) eyes, a V-shaped closure in 14 (31.8%), a flat/open closure in 6 (13.6%), and an irregular closure in 4 (9.1%). We noticed that the best visual recovery was seen in the case of the U-shaped and V-shaped contour of the foveal region (improvement of visual acuity of 3 or more lines), and the irregular closure was observed in 75% of the eyes with ERM preoperatively, although these findings were not statistically significant.

It is well known that postoperative microstructural changes in the macular region involve photoreceptor defects, which may be seen in OCT, correlated with postoperative best-corrected visual acuity (BCVA) [24–26]. In evaluation of OCT changes after macular hole surgery, four distinct hyperreflective lines are crucial: the photoreceptor inner segment/outer segment (IS/OS) junction (referred to by the International Nomenclature OCT Consensus as the ellipsoid zone), the external limiting membrane (ELM), and the cone outer segment tips (COST) [27–29]. Multiple authors reported that the defects in the ellipsoid zone and ELM are a major reason for unsatisfactory postoperative visual recovery [28, 30, 31]. Mitamura et al. suggested that the restoration of the ELM is closely associated with that of the IS/OS junction, and the integrity of the ELM is essential for the restoration of the IS/OS [31]. We have confirmed this observation in our study. In long-term postoperative time, all eyes with ELM defects (19 eyes, 43.2%) had an IS/OS junction defect also. Moreover, all eyes with a normal IS/OS junction line (16 eyes, 63.4%) had a visible ELM line as well. In all of the postoperative examinations, the limiting of the lengths of both the IS/OS junction and ELM defects were significantly correlated with functional improvement of the photoreceptors, measured by visual acuity and microperimetry. We noted slightly better visual acuity in eyes with restored IS/OS junction line, compared to eyes with IS/OS junction defect, which was 0.54 and 0.51, respectively, but these findings were not statistically significant. Similarly, when comparing eyes with a restored ELM line to eyes with an ELM defect, visual acuity was 0.54 and 0.5, respectively. It was also noted in other studies that the restoration of the COST line appears at the latest after MH surgery. The retinal layer becomes restored at the ELM first, followed by the IS/OS, and finally the COST [31, 32]. More cases with a restored ELM line (25 eyes, 56.8%) versus restored IS/OS line (16 eyes, 36.4%) in our study confirm this observation.

Retinal thinning, found in 11 eyes (25%) in early postoperative time and in 14 (31.8%) eyes in the long-term follow-up, was usually combined with a stage-4 MH (72.7% and 64.3%, respectively) and consequently a larger diameter of the MH (>450 *μ*m), which was consistent with the observation of Imamura and Ishida [33]. These eyes had also slightly worse visual acuity in long-term observation. We also noticed that, in 3 (6.8%) eyes with residual cystic cavities, the primary diameter of the MH was over 670 *μ*m, and in postoperative time, the presence of the IS/OS junction defect, ELM defect, and retinal thinning was noted. In these eyes, the preoperative mean visual acuity was worse compared to the whole study group (0.09 versus 0.1) as well as postoperative ones (0.33 and 0.5, respectively), which suggests that eyes with persistent cystic cavities have a worse prognosis in terms of visual and anatomic improvement.

Many studies have shown that after macular surgery, the BCVA improves [16, 34–37]. The operation is considered a functional success if there is a ≥2 Snellen lines (equivalent ≥0.3 logMAR units) improvement [14]. Because of the progressive regeneration of photoreceptors, the recovery of macular function may take time and is usually better after a long-term observation than in short postoperative time. In the multicentre database study in the UK, 48.6% eyes had achieved a success of ≥0.3 logMAR unit improvement at 12 weeks after surgery and 58.3% at 52 weeks [34]. In our study, we achieved the anticipated effect and noted improvement in BCVA between each of the time point examinations—in early postoperative time and in long-term postoperative time (mean BCVA was 0.33 and 0.5, respectively). Visual acuity improvement was achieved in 76.2% eyes up to 6 months after the operation and in 73.8% eyes in the long-term follow-up. These findings were slightly better than in Jackson et al. study but similar to those of Poon et al. in which improvement in the BCVA was noted in 75.6% [15, 34].

We have analysed correlations between preoperative and postoperative changes in the foveal region by assessing OCT scans and visual acuity at different time points. At baseline examination, only proliferations of the RPE were negatively correlated with preoperative visual acuity (*p*=0.03) but had not influenced postoperative BCVA.

Apart from general improvement in visual acuity in the study group, in 9 (21.4%) eyes in early observation and 6 (14.3%) eyes in the long-term one, the BCVA was unchanged, whereas it was lower in 1 (2.4%) eye and 5 (11.9%) eyes, at these time points, respectively. Excluding 2 eyes of 1 patient with considerably decreased BCVA in long-term observation because of the NAION, these percentages are in line with those presented by Meng et al. [36].

A useful tool in functional assessment after MH surgery is colour vision testing. There is a specific anatomic distribution of the L/M- and S-cones, where the L/M-cones are the most densely populated in the foveola, while the S-cones are known to be absent in the foveal region but most noticeable within the central 0.5° [15, 38]. The natural conclusion is that we may suspect more pronounced changes in the red-green axis in central macular diseases such as MH. Poon et al. suggested that, of the 2 chromatic contrast thresholds measured after MH surgery, the red-green contrast threshold (RGCT) showed a significantly stronger correlation with postoperative BCVA improvement than did the Tritan contrast threshold (TCT). However, TCT was significantly elevated (worse) compared with age-matched controls [15]. The improvements in chromatic contrast thresholds correlated with the recovery of the BCVA were also discussed by Madreperla et al. [39]. According to the authors, these improvements are possible by means of the sealing of the MH by Müller cell proliferation and reapproximating edges of the macular hole.

Interesting findings were delivered by von Jagow et al., who assessed the functional outcome of indocyanine green-assisted (ICG) macular surgery [37]. They observed significant changes in colour vision in eyes after ICG-assisted MH surgery, compared to fellow healthy eyes. In the study, they used the Arden colour contrast test index (ACTI), measuring the contrast threshold in percent between the presented optotype and the surrounding area. In the Protan axis, the mean ACTI was 21.7% (with normal range <6%), and in the Tritan axis, the mean ACTI was 48.6% (with normal range <8%), whereas in fellow eyes, the mean ACTI was within normal values in both axes. Relying on the vulnerability hypothesis suggested by Hood et al. that the S-cones are more vulnerable to pathologic processes, we suspect that such profound colour changes in the affected eyes, specifically in the Tritan axis, could be related to the toxicity of the ICG [40].

Anomaloscope examination is based on metameric matching principles. The Rayleigh equation is an important tool in the assessment of acquired colour vision deficiency and is the test of choice for detecting and diagnosing M-L mechanism deficiencies [41]. It enables to identify pseudoprotanomaly and extreme anomalous trichromacy. The equation of zone is within normal limits if *Q* ≤ 1.30 at the green end and *Q* ≥ 0.70 at the red end of the green-red scale. Pseudoprotanomaly is diagnosed if for all the anomaloscope equations, *Q* < 1.0, and the fall in luminance from green to red end of the equation zone is 2 arbitrary scale units at the most. The anomaloscope matches are expressed as the quotient of anomaly (*Q*). The Moreland equation is the most widely used as the Tritan matching function, but it cannot distinguish complete tritanopia from incomplete forms, where residual Tritan colour discrimination is present [42, 43]. These 2 equation methods were combined into a single examination, which assesses acquired colour deficiencies according to their effects on 2 parameters: matching range and midmatching point (MMP) [41, 44]. In our study, such anomaloscopic tests (Rayleigh and Moreland Colour Matching Test) were used. One of the most common mechanisms of acquired colour vision deficiency is an absorption mechanism secondary to the age—associated increase in optical density of the lens pigments [44]. These lens pigments are typically yellow colour and absorb short-wavelength radiation, including ultraviolet and short-wavelength visible light. Consequently, patients usually have worse blue colour perception than other colours. The removal of the crystalline lens causes a shift towards shorter wavelengths. In some patients, shortly after cataract surgery, a cyanopsia occurs. To minimize this effect, yellow intraocular lenses (IOLs) are implanted. Miyata found that the incidence of cyanopsia was higher with clear IOLs than with yellow IOLs [45]. In our study, all of the patients had cataract surgery with implantation of the yellow AcrySof IQ Monofocal IOL a few weeks prior to scheduled vitrectomy, so we assume that cataract surgery was not significant in our evaluation of colour vision outcomes. Tilanus et al. performed anomaloscope examination in macular gliosis, central serous choroidopathy, and macular holes [46]. They used the Nagel II anomaloscope and relied on the rule that diminished red sensitivity indicates for cone involvement with following possible signs: shift of the MMP towards the red end (MMP < 1.0), asymmetrical enlargement of the equation zone towards the red end, and pseudoprotanomaly. In the studied group of 19 eyes with MH, they observed these signs in 21%, 37%, and 16%, respectively. They found that diminished red sensitivity became evident when visual acuity decreased to 0.3 or less, so at an early stage of the MH, with a BCVA > 0.3, there were no evident colour vision abnormalities. Authors from Japan measured the reduction in pigment optical density of cone photoreceptors using the Rayleigh equations in 7 patients with the IF-2 anomaloscope [47]. They noted that, in eyes after macular hole surgery, the Rayleigh equations were shifted toward a protanomalous setting compared to fellow eyes and concluded that recovery of the visual acuity may precede that of the optical density in cone photopigment of central retina. We found the pseudoprotanomaly in 26.3% eyes before the operation and in 35.1% after operation, so our outcomes indicate that anamaloscopically diminished red sensitivity does not improve immediately at postoperative time, confirming observations of the other authors. Although we observed no specific Tritan defects, some changes in the blue-yellow axis, identified by a computer program as an Achromatic Pattern, were noted in most studied eyes, both in preoperative and postoperative time. This raises the question whether they are simply the outcome of concomitant, undetected diseases, or represent the damage to the S-cones on account of mechanical or toxic actions having occurred during the surgical procedure, or perhaps they are caused by a technical error. To answer these questions, we also examined the fellow, “healthy” eyes of 30 patients, primarily scheduled for macular surgery in the other eye. The majority of the patients had very good visual acuity (76.7% of eyes had a BCVA = 1.0). In 7 eyes, we found some abnormalities: VMT in 3 (10%) eyes and initial cataract in 4 (13.3%) eyes. In all these eyes, visual acuity was decreased and vision colour abnormalities in both axes occurred (as pseudoprotanomaly in the Rayleigh equation and an achromatic pattern in the Moreland equation). Other than these 7 eyes, some changes in colour vision were noticeable in completely healthy eyes with a very good BCVA (pseudoprotanomaly was detected in 3 (10%) eyes and an achromatic pattern in 6 (20%) eyes). The most probable reason of such abnormality appears to be the DM. Diabetic retinopathy is commonly associated with acquired colour vision deficiency. In the Early Treatment of Diabetic Retinopathy Study (ETDRS), about 50% of the patients had abnormalities on the Fansworth-Munsell 100-Hue test [48]. There are 2 possible hypotheses of selective S-mechanism deficiency: the scarcity hypothesis and the vulnerability hypothesis [44]. The scarcity hypothesis indicates the central absence of S-cones within the foveal region, so if a pathologic process affects the juxtafoveolar region, a selective S-mechanism deficiency could ensue with comparatively less S-cone loss. Such a mechanism was observed in diabetic patients by Davies and Moreland [49]. The vulnerability hypothesis presumes that there are physiologic and histologic differences between the S-cones and their pathways and the other cones classes, which render the S-cones more vulnerable to pathologic processes [40, 50]. The connection between blue-yellow axis dyschromatopsia preceding diabetic retinopathy was observed in recent studies [51–53]. Gella et al. even stated that acquired impaired colour vision is an early indicator of neurodegenerative changes in the retina and found in diabetic subjects without retinopathy may be of nonvascular etiology [54]. In our study, although only 2 (12.5%) patients from the study group with dyschromatopsia in fellow healthy eye were diagnosed with DM, all of the other 6 patients (20%) had high risk of DM development based on the AGE examination. These observations indicate that other ocular diseases are responsible for some changes in colour vision in seemingly healthy eyes. Some microstructural alterations in the retina, not visible in fundus eye examination or even OCT scans, might occur and influence the function of the photoreceptors.

## 5. Conclusions

In summary, the anatomic and functional outcomes after macular surgery are satisfactory and improve with time. It is consistent with other authors' opinion that after a successfully closed MH, restoration of the retina progresses much slower than improvements in visual acuity. Colour vision evaluation is a useful examination in the assessment of retinal function and may allow to detect certain alterations in the photoreceptors when visual acuity is still good and no changes are detected in the OCT.

## Figures and Tables

**Table 1:** Baseline characteristics of patients (*n*=40) and eyes (*n*=44) that underwent macular hole surgery.

Characteristics	Number of patients and eyes
Age, years	
Mean (±SD)	68.75 ± 6.05
Range	58 to 81

Gender	
Female	28
Male	12

Bilateral MH, number of patients (%)	4 (9.1%)

MH duration, months	
Mean (range)	8.02 (2 to 24)
≤6 months, number of patients (%)	27 (61.4%)
More than 6 months, number of patients (%)	17 (38.6%)

Stage of MH, number of eyes (%)	
Stage 2	5 (11.36%)
Stage 3	17 (38.64%)
Stage 4	22 (50%)

Diameter of MH (*μ*m)	
Mean (range)	468.75 (140 to 790)
≤250 *μ*m	5 (11.36%)
251–450	15 (34.1%)
≥451	24 (54.54%)

ERM, number of eyes (%)	18 (40.9%)

Mean follow- up, months	
Early	≤6
Long-term, mean (range)	18 (9–31)

Operation success (closure of MH), number of eyes (%)	42 (95.45%)

MH, macular hole; ERM, epiretinal membrane.

**Table 2:** OCT changes in eyes before macular surgery, in early and long-term postoperative time.

Before MH surgery		After MH surgery		
OCT changes	Number of eyes (%)	OCT changes	Early follow-up	Long-term follow-up
			Number of eyes (%)	Number of eyes (%)
Cystic cavities	39 (88.6%)	Residual cystic cavities	7 (15.9%)	3 (6.8%)
Elevated edge of the hole	28 (63.6%)	IS/OS junction defects	35 (79.5%)	28 (63.6%)
Proliferations of RPE	38 (86.3%)	ELM defects	27 (61.4%)	19 (43.2%)
		Retinal thinning	11 (25%)	14 (31.8%)

OCT, optical coherence tomography; MH, macular hole; RPE, retinal pigment epithelium; IS/OS junction, photoreceptor inner segment/outer segment junction; ELM, external limiting membrane.

**Table 3:** Preoperative and postoperative visual acuity outcomes (Snellen decimal) in eyes with closed macular holes after surgery.

BCVA	Mean ± SD	Range	Number of eyes	%
Preoperative	0.15 ± 0.13	0.01–0.5	42	100
Postoperative				
Early follow-up (≤6 months)				
Improved	0.33 ± 0.24	0.01–0.8	32	76.2
Unchanged			9	21.4
Decreased			1	2.4
Long-term follow-up (>9 months)				
Improved	0.50 ± 0.32	0.01–1.0	31	73.8
Unchanged			6	14.3
Decreased			5	11.9

BCVA, best-corrected visual acuity.

**Table 4:** Colour vision outcomes in Rayleigh and Moreland equations at 3 time points in affected and fellow eyes.

	Baseline	Early follow-up (≤6 months)	Long-term follow-up (9–31 months)	Fellow eye
Number of eyes	19	15	37	30
Rayleigh equation				
MMP				
Mean ± SD	1.04 ± 0.11	0.98 ± 0.08	0.97 ± 0.08	0.95 ± 0.08
Pseudoprotanomaly (%)	5 (26.3%)	4 (26.7%)	13 (35.1%)	6 (20%)
Moreland equation				
MMP				
Mean ± SD	0.94 ± 0.15	1.0 ± 0.12	0.99 ± 0.11	0.95 ± 0.12
Achromatic pattern (%)	84.2%	93.3%	33 (89.2%)	43.3%

MMP, midmatching point; SD, standard deviation.

## Data Availability

The research article data used to support the findings of this study are included within the article and are available from the corresponding author upon request.

## References

[1] Gass J. D. (2003). Idiopathic senile macular hole: its early stages and pathogenesis. *Retina*.

[2] Kelly N. E., Wendel R. T. (1991). Vitreous surgery for idiopathic macular holes. Results of a pilot study. *Archives of Ophthalmology*.

[3] Eckardt C., Eckardt U., Groos S., Luciano L., Reale E. (1997). Removal of the internal limiting membrane in macular holes. Clinical and morphological findings. *Der Ophthalmologe*.

[4] Lois N., Burr J., Norrie J. (2011). Internal limiting membrane peeling versus no peeling for idiopathic full-thickness macular hole: a pragmatic randomized controlled trial. *Investigative Opthalmology and Visual Science*.

[5] Michalewska Z., Michalewski J., Adelman R. A., Nawrocki J. (2010). Inverted internal limiting membrane flap technique for large macular holes. *Ophthalmology*.

[6] Iwasaki M., Kinoshita T., Miyamoto H., Imaizumi H. (2018). Influence of inverted internal limiting membrane flap technique on the outer retinal layer structures after a large macular hole surgery. *Retina*.

[7] Pichi F., Lembo A., Morara M. (2014). Early and late inner retinal changes after inner limiting membrane peeling. *International Ophthalmology*.

[8] Modi A., Giridhar A., Gopalakrishnan M. (2017). Comparative analysis of outcomes with variable diameter internal limiting membrane peeling in surgery for idiopathic macular hole repair. *Retina*.

[9] la Cour M., Friis J. (2002). Macular holes: classification, epidemiology, natural history and treatment. *Acta Ophthalmologica Scandinavica*.

[10] Meerwaldt R., Graaff R., Oomen P. H. N. (2004). Simple non-invasive assessment of advanced glycation endproduct accumulation. *Diabetologia*.

[11] Stirban A., Heinemann L. (2014). Skin autofluorescence—a non-invasive measurement for assessing cardiovascular risk and risk of diabetes. *European Endocrinology*.

[12] Żuchowski P., Kolossa K., Jeka S. (2015). Assessment of the usefulness of skin autofluorescence as an indicator of disease activity and of the risk of developing diabetes in patients suffering from rheumatoid arthritis. *Reumatologia/Rheumatology*.

[13] de Groot L., Hinkema H., Westra (2011). Advanced glycation end products are increased in rheumatoid arthritis patients with controlled diseases. *Arthritis Research and Therapy*.

[14] Steel D. H., Lotery A. J. (2013). Idiopathic vitreomacular traction and macular hole: a comprehensive review of pathophysiology, diagnosis, and treatment. *Eye*.

[15] Poon W. K., Ong G. L., Ripley L. G., Casswell A. G. (2001). Chromatic contrast thresholds as a prognostic test for visual improvement after macular hole surgery: color vision and macular hole surgery outcome. *Retina*.

[16] Chang E., Garg P., Capone A. (2013). Outcomes and predictive factors in bilateral macular holes. *Ophthalmology*.

[17] Manasa S., Kakkar P., Kumar A., Chandra P., Kumar V., Ravani R. (2018). Comparative evaluation of standard ILM peel with inverted ILM flap technique in large macular holes: a prospective, randomized study. *Ophthalmic Surgery, Lasers and Imaging Retina*.

[18] Narayanan R., Singh S. R., Taylor S. (2018). Surgical outcomes after inverted internal limiting membrane flap versus conventional peeling for very large macular holes. *Retina*.

[19] Byhr E., Lindblom B. (1998). Preoperative measurements of macular hole with scanning laser ophthalmoscopy. Correlation with functional outcome. *Acta Ophthalmologica Scandinavica*.

[20] Amari F., Ohta K., Kojima H. (2001). Predicting visual outcome after macular hole surgery using scanning laser ophthalmoscope microperimetry. *British Journal of Ophthalmology*.

[21] Ullrich S., Haritoglou C., Gass C. (2002). Macular hole size as a prognostic factor in macular hole surgery. *British Journal of Ophthalmology*.

[22] Kusuhara S., Teraoka Escaño M. F., Fujii S. (2004). Prediction of postoperative visual outcome based on hole configuration by optical coherence tomography in eyes with idiopathic macular holes. *American Journal of Ophthalmology*.

[23] Michalewska Z., Michalewski J., Cisiecki S. (2008). Correlation between foveal structure and visual outcome following macular hole surgery: a spectral optical coherence tomography study. *Graefe’s Archive for Clinical and Experimental Ophthalmology*.

[24] Inoue M., Watanabe Y., Arakawa A. (2009). Spectral-domain optical coherence tomography images of inner/outer segment junctions and macular hole surgery outcomes. *Graefe’s Archive for Clinical and Experimental Ophthalmology*.

[25] Baba T., Yamamoto S., Arai M. (2008). Correlation of visual recovery and presence of photoreceptor inner/outer segment junction in optical coherence images after successful macular hole repair. *Retina*.

[26] Haritoglou C., Neubauer A. S., Reiniger I. W. (2007). Long-term functional outcome of macular hole surgery correlated to optical coherence tomography measurements. *Clinical and Experimental Ophthalmology*.

[27] Landa G., Gentile R. C., Garcia P. M., Muldoon T. O., Rosen R. B. (2012). External limiting membrane and visual outcome in macular hole repair: spectral domain OCT analysis. *Eye*.

[28] Staurenghi G., Sadda S., Chakravarthy U., Spaide R. F. (2014). Proposed lexicon for anatomic landmarks in normal posterior segment spectral-domain optical coherence tomography. *Ophthalmology*.

[29] Spaide R. F., Curcio C. A. (2011). Anatomical correlates to the bands seen in the outer retina by optical coherence tomography: literature review and model. *Retina*.

[30] Ruiz-Moreno J. M., Lugo F., Montero J. A. (2012). Restoration of macular structure as the determining factor for macular hole surgery outcome. *Graefe’s Archive for Clinical and Experimental Ophthalmology*.

[31] Mitamura Y., Mitamura-Aizawa S., Katome T. (2013). Photoreceptor impairment and restoration on optical coherence tomographic image. *Journal of Ophthalmology*.

[32] Ooka E., Mitamura Y., Baba T. (2011). Foveal microstructure on spectral-domain optical coherence tomographic images and visual function after macular hole surgery. *American Journal of Ophthalmology*.

[33] Imamura Y., Ishida M. (2018). Retinal thinning after internal limiting membrane peeling for idiopathic macular hole. *Japanese Journal of Ophthalmology*.

[34] Jackson T. L., Donachie P. H. J., Sparrow J. M., Johnston R. L. (2013). United Kingdom National Ophthalmology Database study of vitreoretinal surgery: report 2, macular hole. *Ophthalmology*.

[35] Christensen U. C., Krøyer K., Sander B. (2009). Prognostic significance of delayed structural recovery after macular hole surgery. *Ophthalmology*.

[36] Meng Q., Zhang S., Ling Y. (2011). Long-term anatomic and visual outcomes of initially closed macular holes. *American Journal of Ophthalmology*.

[37] Jagow B. V., Höing A., Gandorfer A. (2009). Functional outcome of indocyanine green-assisted macular surgery: 7-year follow-up. *Retina*.

[38] de Monasterio F. M., McCrane E. P., Newlander J. K. (1985). Density profile of blue-sensitive cones along the horizontal meridian of macaque retina. *Investigative Ophthalmology and Visual Science*.

[39] Madreperla S. A., Geiger G. L., Funata M. (1994). Clinicopathologic correlation of a macular hole treated by cortical vitreous peeling and gas tamponade. *Ophthalmology*.

[40] Hood D. C., Greenstein V. C. (1988). Blue (S) cone pathway vulnerability: a test of a fragile receptor hypothesis. *Applied Optics*.

[41] Roth A., Ohta Y. (1990). The power of metameric color equations in testing color vision. *Color Vision Deficiences*.

[42] Moreland J. D. (2004). Moreland match revisited. *Visual Neuroscience*.

[43] Moreland J. D., Ohta Y. (1990). The clinical utility of anomaloscopy. *Color Vision Deficiencies*.

[44] Simunovic M. P. (2016). Acquired color vision deficiency. *Survey of Ophthalmology*.

[45] Miyata A. (2015). Neutralization method for detecting the incidence of color perception changes after cataract surgery. *Journal of Cataract and Refractive Surgery*.

[46] Tilanus M. A., Pinckers A. J., Aandekerk A. L. (1998). Anomaloscope examination in macular gliosis, macular holes and central serous choroidopathy. *Graefe’s Archive for Clinical and Experimental Ophthalmology*.

[47] Nishio Y., Kandatsu A. (2000). The Rayleigh color matches in idiopathic macular holes treated by vitrectomy. *Japanese Journal of Ophthalmology*.

[48] Fong D. S., Barton F. B., Bresnick G. H. (1999). Impaired color vision associated with diabetic retinopathy: early treatment diabetic retinopathy study report No. 1511A. *American Journal of Ophthalmology*.

[49] Davies N., Moreland A. (2003). Extent of foveal tritanopia in diabetes mellitus. *British Journal of Ophthalmology*.

[50] Greenstein V. C., Hood D. C., Carr R. E. (1989). A comparison of S cone pathway sensitivity loss in patients with diabetes and retinitis pigmentosa. *Documenta Ophthalmologica Proceedings Series*.

[51] Tan N. C., Yip W. F., Kallakuri S., Sankari U., Koh Y. L. (2017). Factors associated with impaired color vision without retinopathy amongst people with type 2 diabetes mellitus; a cross–sectional study. *BMC Endocrine Dosorders*.

[52] Ayed S., Jeddi A., Kallal Z. (1990). Diabetes and color vision disorder detected by the Farnsworth 100 Hue test. Diabetic dyschromatopsia. *Journal Français D’Ophtalmologie*.

[53] Malukiewicz G., Lesiewska-Junk H., Kaźmierczak K. (2009). Changes in the colour vision and contrast sensitivity in diabetic patients without retinopathy. *Klinika Oczna*.

[54] Gella L., Raman R., Kulothungan V., Pal S. S., Ganesan S., Sharma T. (2015). Impairment of colour vision in diabetes with no retinopathy: Sankara Nethralaya diabetic retinopathy epidemiology and molecular genetics study (SNDREAMS-II, report 3). *PLoS One*.

